# Epidemiological and Prognostic Importance of New-Onset Cancer as a Net Adverse Clinical Outcome after ST-Elevation Myocardial Infarction

**DOI:** 10.3390/jcdd11090256

**Published:** 2024-08-23

**Authors:** Toshiharu Fujii, Yuji Ikari

**Affiliations:** Department of Cardiovascular Medicine, Tokai University School of Medicine, Isehara 259-1193, Japan; ikari@is.icc.u-tokai.ac.jp

**Keywords:** ST-elevation myocardial infarction, cancer, mortality, stroke

## Abstract

The study assessed the epidemiological frequency and prognostic impact of new-onset cancer as an additional net adverse clinical outcome in patients after ST-elevation myocardial infarction (STEMI), considering its potential clinical significance alongside classical endpoints. This study was designed as a single-center observational study, including 1285 consecutive patients who were diagnosed as STEMI patients as the subject, and the frequency and prognosis of new-onset cancer after STEMI onset were assessed. The incidence of all-cause death, nonfatal myocardial infarction (MI), stroke, and bleeding were analyzed as classical endpoints. Throughout an average of a 1241.4 days observation period, cancers were observed in 7.0% of patients (n = 90), showing development at a constant rate throughout this period (incidence rate, 0.06/1000 person-years). The average duration from STEMI onset to cancer diagnosis was 1371.4 days. Death, MI, or stroke were observed in 21.3%, 4.0%, 6.5%, and 12.8%, giving incidence rates of 0.18, 0.03, 0.06, and 0.11/1000 person-years, respectively. Long-term mortality was higher in patients with newly diagnosed cancer than in patients without cancer (36.7% vs. 20.1%, *p* < 0.01). Cancer after STEMI should be considered as an additional major adverse clinical event because of its high incidence, constant development, and high mortality in comparison to classical endpoints.

## 1. Introduction

Matured systematic medical intervention has dramatically improved short-term mortality in patients with ST-elevation myocardial infarction (STEMI) [1,2]. However, further effort is still required to improve the associated long-term mortality.

As major adverse clinical endpoints (MACEs), death, myocardial infarction (MI), stroke, and bleeding are considered the most impactful clinical outcomes in both the short- and long-term. However, it has been reported that the major cause of long-term mortality in these patients shifts from cardiac causes of death to noncardiac causes [3], with cancer being described as the primary cause of death after 1 year [4]. Appropriately coping with cancer is indispensable for further improvement in long-term mortality. Nevertheless, the epidemiological and prognostic assessment of new-onset cancer after STEMI is not well-known.

The purpose of the present study was to assess the epidemiological frequency and prognostic impact of new-onset cancer as an additional adverse clinical event after STEMI.

## 2. Materials and Methods

### 2.1. Study Design and Data Source

To assess new-onset cancer after STEMI as a net adverse clinical outcome (NACO), the present study was designed as a single center observational study. The study cohort consisted of STEMI patients admitted to Tokai University School of Medicine from January 2006 to January 2021 with acute symptom onset and with a STEMI diagnosis through emergency coronary angiography. Out of 1286 consecutive patients with STEMI, 1 patient was excluded due to a prior diagnosis of cancer recurrence or metastasis after STEMI. Therefore, a total of 1285 study patients were included in the study cohort. We reviewed the medical records of these patients to investigate new-onset cancer following STEMI during the study period. This study was approved by the Institutional Review Board for Clinical Research of Tokai University Hospital (22R094).

### 2.2. Outcomes and Definitions

STEMI was defined according to the fourth universal definition of myocardial infarction [5]. New-onset cancer, death, nonfatal MI, and nonfatal stroke were assessed as a primary endpoint. The date of new cancer diagnosis was defined as the date of pathological confirmation. Death was defined as an all-cause mortality, including cardiovascular, non-cardiovascular, and undetermined causes. The definition of MI was based on the fourth universal definition of myocardial infarction [5]. Stroke was defined as the sudden onset of a new neurological deficit attributed to an obstruction in cerebral blood flow and/or cerebral hemorrhage with no apparent nonvascular cause confirmed through imaging examination. The severity of illness was assessed at STEMI onset using the GRACE risk score [6]. Bleeding events were defined as Bleeding Academic Research Consortium (BARC) types 3 or 5 [7].

### 2.3. Statistical Analysis

Numerical factors with normal distributions are shown as mean ± standard deviation. Student’s *t*-test was used to determine the significance of differences in clinical parameters between two groups with a normal distribution. The Chi-square test was used to assess the differences in categorical variables. Survival curves and the cumulative incidences of cancer, death, MI, and stroke were plotted using the Kaplan–Meier method. Person-time was calculated as the number of years from the date of STEMI onset to the date of onset of primary endpoints.

The Smirnov–Grubbs test for outliers was applied to the following continuous variables: age, hemoglobin, serum creatinine, estimated glomerular filtration rate (eGFR), and left ventricular ejection fraction (LVEF).

No patients were identified as outliers in age and LVEF. Possible outliers were identified as a hemoglobin value of 4.7 g/dL in 1 patient, platelet counts ranging from 49.2 to 102.3 × 10^4^/μL in 7 patients, serum creatinine levels ranging from 2.16 to 13.5 mg/dL in 57 patients, and eGFRs ranging from 165.8 to 267.3 mL/min/1.73 m^2^ in 3 patients. These values did not require treatment as outliers since they were verified as accurate based on medical records.

The baseline parameters summarized in Table 1 did not include missing values.

*p* values < 0.05 were considered statistically significant. All statistical calculations were performed using STATA statistical software version 14.2 (Stata Corporation, College Station, TX, USA).

## 3. Results

### 3.1. Baseline Characteristics

New-onset cancer after STEMI was evaluated as an adverse clinical event, which are known as NACOs. The baseline characteristics are summarized in Table 1. Over an average observation period of 1241.4 ± 1350.1 days after STEMI onset, new-onset cancer was observed in 7.0% (n = 90) of the patients. The average duration until cancer diagnosis was 1371.4 ± 1174.2 days. A total of 147 patients had their observations censored before reaching any of the primary endpoints. There were no significant differences in baseline parameters between patients with and without new-onset cancer, although the LVEF was higher in patients with cancer compared to those without cancer (55.9% ± 10.3% vs. 51.8% ± 13.0%, *p* < 0.01). The GRACE risk score was similar in the two groups (161.0 ± 36.0 vs. 169.0 ± 50.6, *p* = 0.14).

### 3.2. Incidence of Cancer, Death, MI, Stroke, and Bleeding

**Figure 1:**
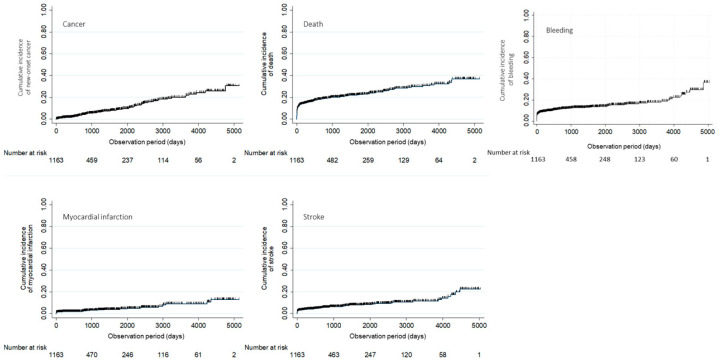
Cumulative incidences of cancer, death, MI, stroke, and bleeding.

**Figure 2:**
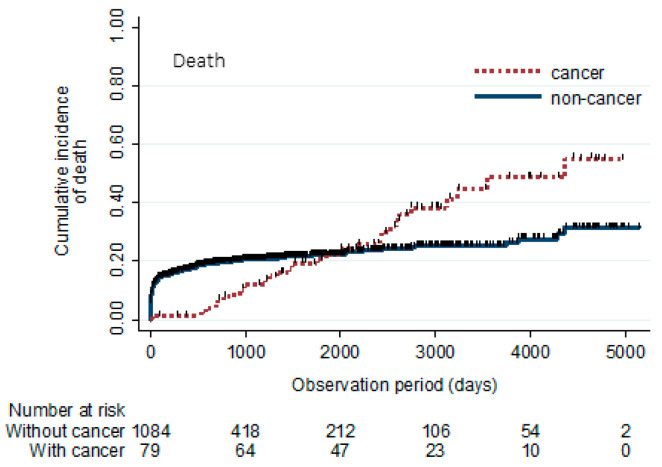
Survival curves in patients with and without cancer.

The cumulative incidences of cancer, death, MI, stroke, and bleeding were 7.0%, 21.3%, 4.0%, 6.5%, and 12.8%, respectively (Figure 1 and Figure 2). These incidence rates were 0.06 per 1000 person-years for cancer (a total of 1,295,551 person-time), 0.18 per 1000 person-years for death (a total of 1,368,264 person-time), 0.03 per 1000 person-years for MI (a total of 1,320,518 person-time), 0.06 per 1000 person-years for stroke (a total of 1,307,699 person-time), and 0.11 per 1000 person-years for bleeding (a total of 1,298,483 person-time).

New-onset cancer after STEMI showed a constant increment throughout the observation period. Cancer: incidence rate, 0.06 per 1000 person-years; total, 1,295,551 person-time. Death: incidence rate, 0.18 per 1000 person-years; total, 1,368,264 person-time. MI: incidence rate, 0.03 per 1000 person-years; total, 1,320,518 person-time. Stroke: incidence rate, 0.06 per 1000 person-years; total, 1,307,699 person-time. Bleeding: incidence rate, 0.11 per 1000 person-years; total, 1,298,483 person-time.

Cancer origins were as follows: lower gastrointestinal (25.6%), lung (20.0%), upper gastrointestinal (13.3%), prostate (11.1%), urinary tract (7.8%), biliary (4.4%), liver (4.4%), hematological (3.3%), craniocervical (3.3%), breast (2.2%), and others (4.4%).

The survival curves of patients with and without cancer are illustrated in Figure 2. Mortality was lower in patients with cancer than in those without cancer in the early phase. However, this relationship reversed in the late phase (36.7% vs. 20.1%, *p* < 0.01).

The survival curves after STEMI in patients with and without cancer are shown. Although mortality is lower in patients with cancer than in those without cancer in the early phase, the relationship reverses after about 2000 days.

## 4. Discussion

The present study assessed the epidemiological frequency and prognostic impact of new-onset cancer as an additional adverse clinical event after STEMI. Cancer incidence increased steadily over time, affecting 7.0% of patients during observation period (0.06 per 1000 person-years). Cancer incidence exceeded that of MI and was comparable to stroke. Although early mortality was lower in the cancer group, the cumulative deaths in the cancer group surpassed those in the non-cancer group in the later phase.

Net adverse clinical events (NACEs), encompassing death, MI, stroke, and bleeding, have traditionally served as the primary outcome measure in post-STEMI patients due to their profound influence on mortality and quality of life. However, the contemporary era of prompt primary percutaneous coronary intervention has witnessed a shift in the leading cause of long-term mortality, with a transition from cardiac to non-cardiac deaths [3]. This decline in cardiac mortality is attributed to advancements in medical interventions during both acute and chronic phases, resulting in a decrease in deaths from MI and sudden cardiac death. Conversely, the rise in non-cardiac mortality is linked to an increase in cancer-related deaths [3]. While cardiogenic shock remains the dominant cause of death within the first 30 days post-STEMI, cancer assumes the top position beyond the one-year mark [4]. Given its substantial impact on mortality and quality of life, comparable to the classical NACEs, cancer warrants a prominent place as a critical clinical outcome, with its clinical importance potentially equaling or surpassing classical NACEs.

Bleeding is another concern in patients with MI who have undergone intervention with current drug-eluting stents. Dual antiplatelet therapy (DAPT) is the standard treatment for preventing thrombosis but is also associated with a significant risk of bleeding. By assessing bleeding risk using ARC-HBR, it is crucial to carefully balance the risk of thrombotic events against the risk of bleeding complications through strategies such as shortening DAPT duration or selecting appropriate P2Y12 receptor inhibitors [8,9,10].

The Danish National Prescription Registry suggested the possibility that MI raises the risk of cancer. Out of 122,275 patients after MI, 11,375 developed cancer over 16 years, compared to 372,397 cases of cancer observed in a reference population of 2,748,893 (hazard risk 1.14, 95% CI 1.09 to 1.20) [11]. The Tromsø Study, which involved 30,586 healthy subjects in a population-based study with repeated health surveys in Norway, found that 1747 subjects developed incident MI out of the general population. Among these, 146 individuals experienced a subsequent cancer during a median follow-up of 15.7 years. MI was revealed to significantly increase the risk of incident cancer even after adjusting for confounders [12]. Several reports have investigated the underlying mechanism. The primary reason cited is the shared risk factors between coronary disease and cancer [11,13,14,15]. Heart failure has been associated with an increased risk of cancer [16], possibly due to oxidative stress [17,18,19]. While constant medical care following MI may increase the likelihood of cancer diagnosis, cardiovascular medications such as angiotensin-receptor blockers, cardiac glycosides, diuretic agents, and statins have been shown not to increase the risk of cancer [16,20,21,22].

A long-term follow-up study spanning approximately 50 years of heart disease development in men aged 40–59 found that malignancies were the leading cause of death (40.4%) among those who did not develop coronary artery disease or other heart conditions. Among those who did develop coronary artery disease or other heart diseases, malignancies accounted for 12.7% and 15.7% of deaths, respectively [23]. Conversely, observational studies of cancer survivors have shown an increased risk of cardiovascular events (crude risk ratio 1.07, 95% CI: 1.04–1.11, *p* < 0.01; adjusted risk ratio 1.02, 95% CI: 0.99–1.06, *p* = 0.17). Notably, individuals with multiple myeloma, lung/bronchus carcinoma, non-Hodgkin lymphoma, or breast cancer had significantly higher risks of cardiovascular events [24]. A meta-analysis by Li et al. (2019) suggested the following: (i) an increased risk of cancer was observed only in women, (ii) the risk of cancer incidence was high in the first six months but became non-significant with increasing follow-up duration, and (iii) there is a possibility that shared risk factors contribute to a higher incidence of cancer. Based on these findings, they concluded that the link between MI and cancer incidence remains unclear [25]. A population-based cohort study using a nationwide database in Taiwan reported that the risk of newly diagnosed breast cancer within approximately five years after myocardial infarction was not associated with myocardial infarction. However, the investigation of risk factors in this Taiwanese study was limited due to the use of a large government-operated administrative claims database, which excluded existing myocardial infarction patients [26]. Hence, concerns about selection bias exist, and these findings also suggest the link between MI and cancer development remains controversial.

In contrast to classical NACEs, which are concentrated in the acute phase, cancer incidence demonstrated a steady increase throughout the observation period. Mortality from cancer also increased steadily in parallel with the increasing cancer incidence. Notably, approximately 2000 days after STEMI onset, the relationship between cumulative death incidence reversed between the groups with and without cancer. The prognostic impact of cancer is as significant as that of the classical NACEs. Therefore, we recommend paying attention to new-onset cancer in routine clinical practice and suggest regular cancer screening for patients after MI.

In the present STEMI cohort, the lower gastrointestinal tract and lungs were identified as the most common primary sites of cancer. Primary origins are dominant in the lower and upper gastrointestinal tract (prostate and upper gastrointestinal tract in males; lower gastrointestinal tract and lung in females) in the general Japanese population [27], and breast and prostate in a report from Danish national registries [11]. Although these variations in primary organs depend on the age, gender, or race of the survey cohort, we hypothesize that MI does not increase the risk in all organs. One report suggested that the frequency of cancer occurrence varies among different organs in patients after MI, with high incidence rate ratios observed in the lung and lower urinary tract [11]. The difference in the impact of cancer development between each organ has not been well discussed. If it differs from the healthy cohort, we should provide a specific cancer screening protocol for patients after STEMI, unlike a general screening protocol for a healthy cohort. Conducting a long-term follow-up survey on a large cohort consisting of patients with and without STEMI is necessary to confirm this hypothesis. Furthermore, in the cancer group of the present study cohort, a male dominance was observed, which is in contrast to the general trend of female dominance in cancer [12]. It is necessary to assess the impact of MI on gender differences in cancer incidence.

The interpretation of the present study may be limited by several factors. The group with cancer likely experienced fewer early deaths because these patients had to survive until they received a cancer diagnosis. In other words, individuals who died early were less likely to be diagnosed with cancer and were therefore excluded from the cancer group, leading to a lower early mortality rate in this group. The group without cancer might have included patients with undiagnosed cancer who died before their cancer was discovered. This potential confounding factor should be considered when interpreting differences in early-phase mortality. Despite these limitations, the data from this study provide valuable real-world evidence.

The present study has several limitations. First, it was conducted as an observational study, which limits the ability to draw causal inferences. Second, the observation period was short in some patients due to loss to follow-up, which may have introduced bias into the results. Third, the data were obtained from a single center, which limits the generalizability of the findings.

## 5. Conclusions

New-onset cancer after STEMI should be considered an important adverse clinical event, which are known as NACOs. Our data showed that a certain proportion of patients developed cancer following STEMI onset. Cancer occurred more frequently than MI and at a rate similar to that of stroke. It was associated with high mortality.

## Figures and Tables

**Table 1:** Baseline characteristics.

	Overall n = 1285	With Cancer n = 90	Without Cancer n = 1195	*p* Value
Age, years	66.8 ± 12.6	67.3 ± 9.9	66.8 ± 12.7	*0.67*
Male, n	1011 (78.7%)	77 (86.0%)	934 (78.0%)	*0.11*
Hypertension, n	965 (75.1%)	60 (66.7%)	905 (75.7%)	*0.06*
Dyslipidemia, n	874 (68.0%)	65 (72.2%)	809 (67.7%)	*0.41*
Diabetes mellitus, n	451 (35.1%)	31 (34.4%)	420 (35.1%)	*1.00*
Insulin, n	68 (5.3%)	5 (5.6%)	63 (5.3%)	*0.81*
Smoking history, n	841 (65.5%)	67 (74.4%)	774 (64.8%)	*0.06*
Prior cancer, n	110 (8.6%)	8 (8.9%)	102 (8.5%)	*0.91*
Hemoglobin, g/dL	14.1 ± 2.3	14.0 ± 2.3	14.2 ± 2.3	*0.45*
Platelet, ×10^4^/μL	21.9 ± 11.2	20.9 ± 5.6	22.0 ± 11.5	*0.39*
Serum creatinine, mg/dL	1.2 ± 1.3	1.0 ± 0.8	1.2 ± 1.3	*0.27*
eGFR, mL/min/1.73 m^2^	61.9 ± 23.5	66.1 ± 21.9	61.6 ± 23.6	*0.08*
Hemodialysis, n	29 (2.3%)	1 (1.1%)	28 (2.3%)	*0.72*
LVEF, %	52.1 ± 12.9	55.9 ± 10.3	51.8 ± 13.0	*<0.01*
GRACE risk score	168.5 ± 49.7	161.0 ± 36.0	169.0 ± 50.6	*0.14*

eGFR = estimated glomerular filtration rate; LVEF = left ventricular ejection fraction.

## Data Availability

The datasets presented in this article are not available due to the requirement for acceptance by the Institutional Review Board for Clinical Research of Tokai University Hospital.
